# 产科弥散性血管内凝血临床诊断与治疗中国专家共识

**DOI:** 10.3760/cma.j.issn.0253-2727.2023.08.002

**Published:** 2023-08

**Authors:** 中华医学会血液学分会血栓与止血学组

产科弥散性血管内凝血（disseminated intravascular coagulation, DIC）是在产科疾病基础上发生的，以出血、栓塞及微循环障碍为特征的临床病理综合征。总体发生率为0.03％～0.35％[1]–[2]。胎盘早剥、羊水栓塞、HELLP综合征、围产期脓毒症、死胎滞留等是并发产科DIC的常见疾病[2]。产科原发病发展到DIC的过程隐匿，缺乏预警。早期识别、正确诊断和及时处理至关重要。本共识由产科及血液科出凝血专家就上述常见产科DIC的诊断、实验室检查和处理给出推荐，供广大临床医务人员参考，以降低孕产妇和胎婴儿死亡率、改善妊娠结局。

一、产科DIC的发病机制

妊娠期表现为生理性高凝状态[3]，血液循环中凝血因子Ⅶ、Ⅷ、Ⅸ、Ⅹ水平明显升高，纤维蛋白原（Fib）水平为未孕时的2倍，达到4～6 g/L[1],[4]，抗凝系统蛋白S水平明显下降，D-二聚体和纤维蛋白（原）降解产物（FDP）随孕周增加而升高，胎盘来源的纤溶酶原激活物抑制剂表达上升，组织型纤溶酶原激活物水平降低[5]。

组织因子是产科DIC凝血激活的主要始动环节。胎盘、蜕膜、子宫肌层、羊水富含组织因子，含量分别是血浆中的数十到上千倍[6]。发生严重胎盘早剥、羊水栓塞、宫内感染致脓毒症、死胎滞留时[7]–[8]，大量组织因子进入母体血液循环，通过凝血级联激活Fib，形成纤维蛋白，微血栓广泛生成；继而激活纤溶系统，继发纤维蛋白溶解亢进，从而导致广泛出血及器官功能障碍。

二、产科DIC的临床表现

产科DIC的临床表现因原发病不同而差异很大，主要表现如下：

1. 出血：表现为产后大出血且血液不凝、腹膜后间隙广泛渗血、手术缝合止血困难；全身皮肤、黏膜出血（穿刺部位出血、消化道自发出血、尿血等）。

2. 休克：出现低血压、低血氧、少尿或无尿等休克表现。并发DIC时，休克不易纠正。

3. 微血管栓塞：发生于器官的微血管栓塞其临床表现各异，可表现为呼吸衰竭、意识障碍、肝肾功能衰竭等，严重者可导致多器官功能衰竭。

4. 微血管病性溶血：表现为微血管病性溶血性贫血，贫血程度与出血量不成比例，偶见皮肤黏膜黄染。

三、产科DIC的实验室检查

凝血酶原时间（PT）和活化部分凝血活酶时间（APTT）、Fib浓度及血小板计数反映了凝血因子消耗，FDP、D-二聚体反映纤溶系统活化，推荐上述指标作为DIC的主要诊断指标。妊娠期存在生理性高凝状态，需非常重视上述指标的动态变化。

四、诊断

妊娠期DIC的诊断主要依据原发病、临床表现、实验室检查，强调综合分析和动态监测。目前单一实验室指标尚不能实现产科DIC的早期特异性诊断，在临床工作中，当检测结果回报时，患者病情往往发生了显著变化。DIC评分系统兼顾临床表现及实验室指标动态变化，具有显著的诊断效能，因此对于产科DIC的早诊早治具有重要意义。本共识参考2017年中华医学会血液学分会血栓与止血学组发表的中国弥散性血管内凝血诊断积分系统（Chinese DIC scoring system, CDSS）[9]，制定产科弥散性血管内凝血诊断评分表（表1），若总计评分超过7分，可诊断DIC。

**表1:** 产科弥散性血管内凝血诊断评分表

评分项	分数
产科原发病	2
胎盘早剥；羊水栓塞；HELLP综合征；宫内感染；死胎滞留	
临床表现	
出血倾向：产后大出血止血困难，产道或手术创面出血、渗血、止血困难，肉眼可见血尿和黑便，紫癜，黏膜出血，齿龈出血，注射部位出血等	1
多器官脏器功能衰竭：少尿或无尿，急性呼吸功能衰竭，肺部啰音、粉红泡沫痰等心衰表现，肉眼可见黄疸，意识模糊，抽搐，坏死性肠炎、其他重要器官衰竭	1
休克：脉搏超过100次/min或血氧饱和度（SpO_2_）≤90%、血压下降超过40%、冷汗、苍白	1
实验室指标	
血小板计数	
≥100×10^9^/L	0
（80~<100）×10^9^/L	1
<80×10^9^/L	2
24 h内下降≥50%	1
D-二聚体	
<5 mg/L	0
（5~<9）mg/L	2
≥9 mg/L	3
凝血酶原时间（PT）及活化部分凝血活酶时间（APTT）延长	
PT延长<3 s且APTT延长<10 s	0
PT延长≥3 s或APTT延长≥10 s	1
PT延长≥6 s	2
纤维蛋白原	
≥1.0 g/L	0
<1.0 g/L	1

五、产科DIC的特征性表现

1. 胎盘早剥并发DIC：表现为出血不凝、血尿、严重时出现休克、肾功能衰竭等，胎盘娩出后见母体面大面积凝血块压迹。实验室检查常出现D-二聚体、FDP升高、Fib水平进行性下降[10]，PT大多正常，血小板计数可下降。

2. 羊水栓塞并发DIC：羊水栓塞临床表现多样，70％的羊水栓塞发生在产程中，11％发生在经阴道分娩后，19％发生于剖宫产术中及术后。典型临床表现为产时、产后突发的低氧血症、低血压和凝血功能障碍[11]。常表现为产后出血、术中创面出血不凝，血标本凝固或溶血而无法检测。休克与出血量不成正比，易出现肾功能衰竭、中枢神经系统损害等多器官受累表现。临床上较难捕捉到高凝期，病情迅速进展进入低凝期。

3. 重度子痫前期/HELLP综合征并发DIC：重度子痫前期并发HELLP综合征患者中DIC的发生率约为15％[12]，主要发生于严重微血管病性溶血病例中，孕妇表现为高血压、水肿、头痛、视物模糊等主要临床表现，轻度黄疸。实验室检查LDH明显升高，胆红素轻度升高、网织红细胞增加，血小板下降较为显著[13]。

4. 妊娠期和产褥期脓毒症并发DIC：脓毒症性DIC可有发热、寒战、神志改变、呕吐、皮疹、呼吸困难、腹痛、腹泻、全身乏力、休克等症状。实验室检查特点包括血小板减少或进行性下降，PT、APTT延长，D-二聚体、FDP逐渐增加，Fib降低不显著[14]–[15]。

5. 死胎滞留并发DIC：通常进展缓慢，是持续亚临床激活凝血级联反应的过程。胎儿死亡后2～3周开始出现Fib的减少，随着滞留时间的延长，Fib的消耗程度逐渐增加，凝血因子Ｖ、Ⅶ含量下降，血小板数减少，D-二聚体、FDP增加[16]–[17]。临床上因我国围产保健的加强，超声的应用，死胎往往会被及时发现，长期滞留情况较少。

六、鉴别诊断

产科DIC与产后出血、妊娠期急性脂肪肝、TTP-HUS综合征易发生混淆。

1. 产后出血并发稀释性凝血病：严重产后出血时，大量快速凝血因子和血小板丢失，液体复苏加重血液稀释，出现类似于DIC的止凝血指标变化。但产科出血本身并不是造成DIC的原因，最终的凝血功能障碍并非高凝栓塞继发纤溶导致，其特点为消耗和稀释。

2. 妊娠急性脂肪肝并发凝血功能障碍：妊娠急性脂肪肝常伴肝功能衰竭，凝血因子合成障碍，导致凝血功能异常[12]。起病初期血浆Fib水平即明显低下，易并发产后出血。

3. 血栓性血小板减少性紫癜（thrombotic thrombocytopenic purpura, TTP）和溶血性尿毒症（hemalytic-uremic syndrome, HUS）：TTP和HUS均属于血栓性微血管病，主要病理特征是血管内皮细胞损伤后引起微动脉和毛细血管微血栓形成，有溶血及肾脏受累的症状[18]–[19]。与DIC不同，TTP和HUS患者虽然存在溶血和血小板降低，但其他凝血指标如PT、APTT、Fib正常。因纤溶不亢进，故D-二聚体、FDP不升高。

七、治疗

治疗原则：积极治疗产科原发病，同时根据实验室检查，补充血液制品，纠正凝血功能保护重要脏器[20]。

1. 原发病治疗：产科DIC治疗的根本在于病因处理，尽快终止妊娠，减少组织因子入血是关键[13]。难治性产后出血积极抢救无效考虑子宫切除术。脓毒症时做好液体复苏，尽快使用广谱抗生素，评估和监测胎儿健康状态酌情终止妊娠[21]。

2. 抗凝治疗：抗凝治疗的目的是阻止凝血过度活化，主要用于产科DIC早期高凝状态。但当患者出现明显临床表现时，往往机体已进入凝血因子消耗期。例如羊水栓塞的高凝过程非常短暂，当发生产后出血、或者器官功能障碍时已经失去抗凝时机。肝素在活动性出血、凝血因子缺乏及纤溶亢进者不宜使用。对于胎盘早剥、HELLP综合征、妊娠合并感染等情况，在终止妊娠后，为避免高凝状态发生血栓，可预防性使用低分子肝素防治深静脉血栓的发生[22]。

3. 补充血液制品：产科DIC发生活动性出血，建议输血维持HGB 70～80 g/L[23]，对于稳定生命体征非常重要。当Fib<1.5 g/L，应用0.1～0.15 U/kg冷沉淀；也可使用浓缩纤维蛋白原4～6 g，维持Fib 2 g/L以上。当PT、INR及APTT超过正常值上限1.5倍而仍有活动性出血时，建议输入新鲜冰冻血浆10～20 ml/kg[24]。当血小板计数低于50×10^9^/L伴活动性出血时，可紧急输注血小板[24]–[25]。

4. 重组人凝血因子Ⅶa：可通过与组织因子结合，促进凝血酶的形成而达到迅速止血的目的[26]。

5. 其他治疗

（1）支持对症：保暖、抗休克、纠正缺氧、酸中毒及电解质紊乱。

（2）纤溶抑制剂：对于DIC的患者一般不推荐使用抗纤溶治疗[27]。但对于羊水栓塞引起的产后出血，可使用氨甲环酸预防继发纤溶亢进[11]。注意应尽早使用，当已发生DIC时使用，须警惕栓塞的发生。

（3）糖皮质激素：羊水栓塞时尽早使用大剂量糖皮质激素（氢化可的松、甲泼尼龙等）[11]。

（4）抗生素：围产期脓毒症尽早针对性应用抗生素。

（5）必要时应用体外膜氧合器（ECMO）、血浆置换处理多器官功能衰竭。
